# Sphingolipid profiling reveals differential functions of sphingolipid biosynthesis isozymes of *Caenorhabditis elegans*

**DOI:** 10.1016/j.jlr.2024.100553

**Published:** 2024-05-03

**Authors:** Hui Luo, Xue Zhao, Zi-Dan Wang, Gang Wu, Yu Xia, Meng-Qiu Dong, Yan Ma

**Affiliations:** 1 Chinese Academy of Medical Sciences & Peking Union Medical College, Beijing, China; 2 National Institute of Biological Sciences (NIBS), Beijing, China; 3 Tsinghua Institute of Multidisciplinary Biomedical Research, Tsinghua University, Beijing, China; 4 College of Chemistry and Chemical Engineering, Inner Mongolia University, Hohhot, China; 5 MOE Key Laboratory of Bioorganic Phosphorus Chemistry & Chemical Biology, Department of Chemistry, Tsinghua University, Beijing, China

**Keywords:** *Caenorhabditis elegans*, mass spectrometry, sphingolipid, ceramide, glucosylceramide, sphingomyelin, monomethyl branched chain fatty acid

## Abstract

Multiple isozymes are encoded in the *Caenorhabditis elegans* genome for the various sphingolipid biosynthesis reactions, but the contributions of individual isozymes are characterized only in part. We developed a simple but effective reversed-phase liquid chromatography-tandem mass spectrometry (RPLC-MS/MS) method that enables simultaneous identification and quantification of ceramides (Cer), glucosylceramides (GlcCer), and sphingomyelins (SM) from the same MS run. Validating this sphingolipid profiling method, we show that nearly all 47 quantifiable sphingolipid species found in young adult worms were reduced upon RNA interference (RNAi) of *sptl-1* or *elo-5*, which are both required for synthesis of the id17:1 sphingoid base. We also confirm that HYL-1 and HYL-2, but not LAGR-1, constitute the major ceramide synthase activity with different preference for fatty acid substrates, and that CGT-3, but not CGT-1 and CGT-2, plays a major role in producing GlcCers. Deletion of *sms-5* hardly affected SM levels. RNAi of *sms-1, sms-2,* and *sms-3* all lowered the abundance of certain SMs with an odd-numbered N-acyl chains (mostly C21 and C23, with or without hydroxylation). Unexpectedly, *sms-2* RNAi and *sms-3* RNAi elevated a subset of SM species containing even-numbered N-acyls. This suggests that sphingolipids containing even-numbered N-acyls could be regulated separately, sometimes in opposite directions, from those containing odd-numbered N-acyls, which are presumably monomethyl branched chain fatty acyls. We also find that ceramide levels are kept in balance with those of GlcCers and SMs. These findings underscore the effectiveness of this RPLC-MS/MS method in studies of *C. elegans* sphingolipid biology.

Sphingolipids are among the most abundant classes of membrane lipids in eukaryotes, second only to glycerophospholipids (1, 2). Distinct from glycerophospholipids, which are built from a glycerol-3-phosphate backbone, sphingolipids have a backbone of long-chain aliphatic amine, typically with zero or one double bond between C4 and C5, one amine group (C2), and a variable number of OH groups (C1, C3, and C4). (3). This sphingoid base is synthesized from long-chain fatty acyl-CoA and serine. In mammals, palmitoyl-CoA is often used as a substrate and the resulting sphingoid base is typically d18:1 (d denotes the two -OH groups, 18 is the total number of carbon atoms, and 1 is the number of double bond) (4).

Among three classes of sphingolipids—ceramides, glycosylceramides, and SMs—ceramides have the simplest structure with only a fatty acyl chain attached to the C2 amine group. Ceramides are synthesized on the endoplasmic reticulum (ER) membrane (5) and further transferring to the ceramide C1 hydroxyl group of sugar moieties on the *cis* and medial Golgi membrane (6, 7) or of phosphocholine on the *trans* Golgi membrane (8) generates glycosylceramides or SMs, respectively.

As structural components of cell membranes, SMs are highly enriched in the outer leaflet of the plasma membrane (PM) (9), while ceramides and glycosylceramides are also present in high abundance in the endomembrane system in addition to the PM on both leaflets (8, 10, 11). By concentrating membrane receptors and signaling molecules, sphingolipid membrane microdomains—also known as lipid rafts—provide a central platform for efficient transduction of cellular signals (12). Sphingolipids and their metabolites also serve as second messengers to regulate cell growth, differentiation, and cell death (11, 13, 14). Dysregulation of sphingolipid metabolism has been linked to many human diseases, including neurodegenerative conditions (15), type II diabetes (16), cardiovascular diseases (17), and cancers (18).

Sphingolipids of the nematode *Caenorhabditis elegans* are rather peculiar compared to their mammalian counterparts (19, 20). The sphingoid base of *C. elegans*, from which other worm sphingolipids such as ceramides, glycosylceramides, and SMs are derived, is predominantly id17:1 (83%), followed by a minor alternative id17:0 (12%) and trace amounts of others (20, 21) (Fig. 1A). The two C17-iso-branched sphingoid bases (id17:0/id17:1) are modified condensation products of serine and C15iso—a monomethyl branched chain fatty acid (mmBCFA) (22, 23) (Fig. 1A). The glycosylceramides of *C. elegans* are found to be predominantly glucosylceramides (GlcCers) and contain 2-hydroxylated N-acyl chains (19, 24). For *C. elegans*’ ceramides and SMs, both hydroxylated and non-hydroxylated N-acyl chains are found (24). Like their mammalian counterparts, the N-acyl chains of worm sphingolipids are primarily saturated, long or very long chain fatty acids (25).

**Fig. 1:**
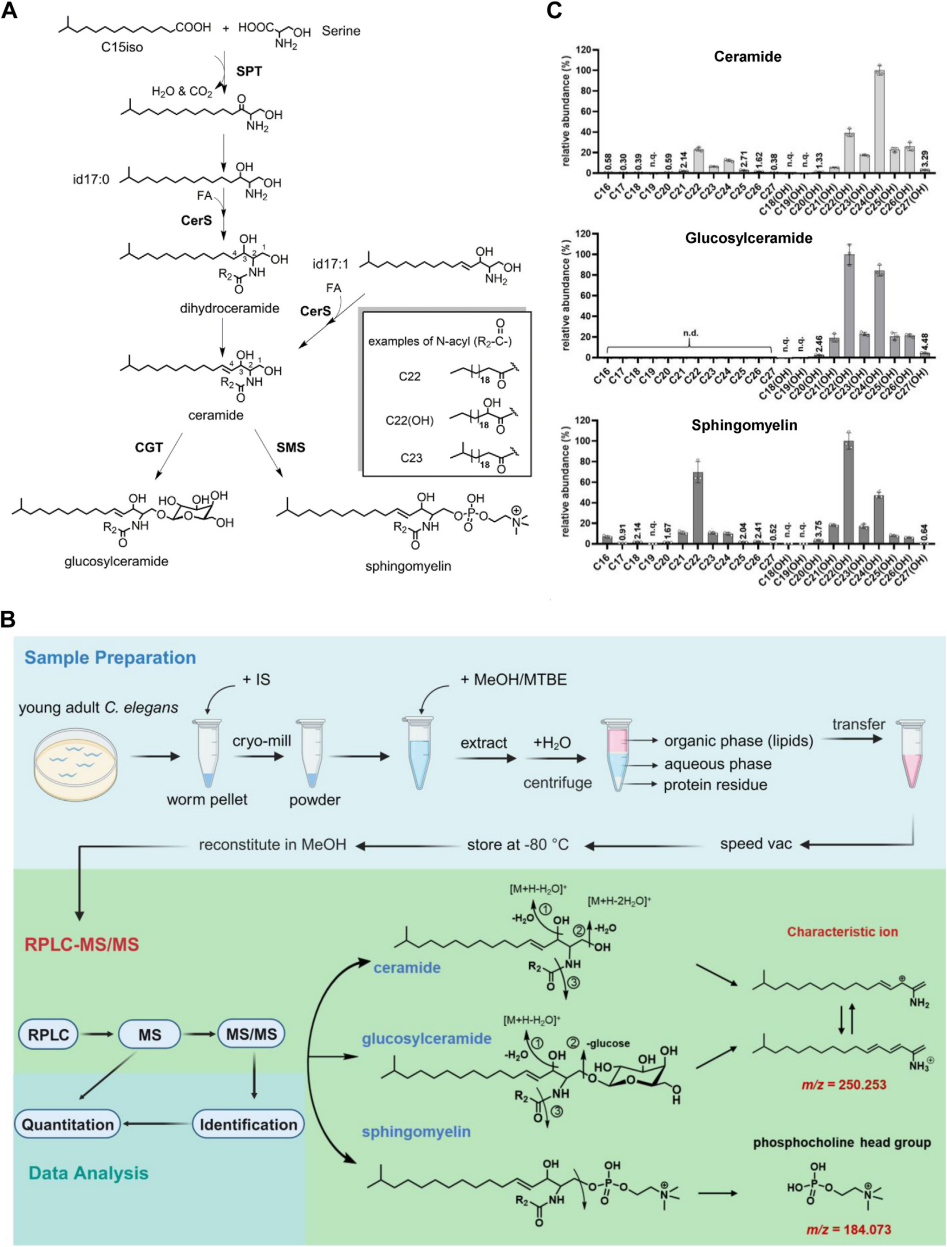
Establishment of a RPLC-MS/MS workflow for the analysis of *Caenorhabditis elegans* sphingolipids. A: A diagram of the de novo sphingolipid biosynthesis pathway in *C. elegans*. Serine palmitoyltransferase (SPT) catalyzes the condensation of a monomethyl branched chain fatty acid C15ISO and serine to form the precursor of the iso-branched d17:1 (id17:1) sphingoid base. Ceramide synthase (CerS) catalyzes the acylation of a sphingoid base to form ceramide (Cer) or dihydroceramide. A ceramide glucosyltransferase (CGT) and a sphingomyelin synthase (SMS) transfers a glucose or a phosphocholine head group to the C1 hydroxyl of ceramide to form glucosylceramide (GlcCer) or sphingomyelin (SM), respectively. B: The workflow for sphingolipid profiling. Created with BioRender.com. Total lipids were extracted from 100 μl of tightly packed young adult *C. elegans.* The accurate mass of the precursor ion and that of the characteristic fragment ions were used to identify sphingolipids, *m/z* 250.253 for Cer and GlcCer and *m/z* 184.073 for SM. C: A total of 54 sphingolipids were identified from WT *C. elegans*. n.d. (not detected), n.q. (not quantifiable). The highest intensity species of each subclass was set to 100% relative abundance as a reference to scale other species of the same subclass.

Sphingolipids are essential for *C. elegans*; mutants that cannot synthesize sphingolipids arrest as L1 larvae and die (22, 26, 27, 28). The underlying molecular mechanism has been unveiled through a series of investigations and GlcCer is found to be an essential growth signal that activates mammalian target of rapamycin complex 1 (mTORC1) in *C. elegans* (27, 29, 30). Additionally, mutants lacking GlcCer fail to establish apicobasal polarity of the gut epithelial cells and suffer indigestion and larval arrest (10, 29). The localization of membrane proteins can also be affected when GlcCer synthesis is disrupted (31). A specific C22-GlcCer is reported as a metabolite associated with longevity (32). Concerning other sphingolipids, ceramides are required for the anoxia response (33) as well as radiation-induced germline apoptosis in *C. elegans* (34), and SMs with very long-chain fatty acids (VLCFAs) are implicated in the cuticle barrier function (35).

*C. elegans* worms cultured in the laboratory must synthesize sphingolipids on their own since their *E. coli* diet contains no sphingolipids (36, 37). The *C. elegans* genome encodes a full set of enzymes required for the biosynthesis of sphingolipids, including multiple isozymes whose functions are characterized incompletely. Specifically, there are three ceramide synthases (HYL-1, HYL-2, LAGR-1) (33, 34, 38, 39), three UDP-glucose:ceramide glucosyltransferases (CGT-1, -2, and -3) (27) for the synthesis of GlcCers, and four SM synthases (SMS-1, -2, -3, and -5). According to the wormbase annotation, SMS-5 is a homolog of human SMS1, which is mainly localized to the Golgi where de novo synthesis of SMs takes place (40). SMS-1, -2, and -3 are homologs of human SMS2, which is localized to the Golgi membranes as well as the PM (8, 40), where it can remodel SMs by resynthesizing them from ceramides generated by SMases.

Except for HYL-1 and HYL-2 (33), the substrate specificity or product profiles of the above isozymes have not been characterized, as previous studies were concerned mainly with genetic analysis of developmental phenotypes (27, 41).

In this study, we established a reversed-phase liquid chromatography-tandem mass spectrometry workflow for rapid and sensitive quantitation of *C. elegans*’ ceramides, GlcCers, and SMs all at once. In total, 54 sphingolipids were identified and 47 quantified from young adult worms. We validated this workflow using *C. elegans* worms in which the key enzymes responsible for generating the C15iso mmBCFA (*elo-5*) or the sphingoid base (*sptl-1*) were knocked down. Application of this workflow to isozyme characterization revealed rich information about the ceramide synthases, CGTs, and SM synthases. For the ceramide synthases, *hyl-1* or *hyl-2* RNA interference (RNAi) markedly reduced the abundance of most or all ceramide species, whereas *lagr-1* RNAi or mutation had only a weak effect. Regarding the CGTs, deficiency of *cgt-3* but not *cgt-1* or *cgt-2* markedly decreased the abundance of all GlcCers quantified in *C. elegans*. Among the three worm homologs of human SMS2, none affected all SMs when knocked down, but each reduced the abundance of a subset of SMs. Deletion of *sms-5*, the sequence homolog of human SMS1, did not reduce any SMs. Our data also show that reduction of a sphingolipid subclass (or a subset of species within a subclass), as a direct result of RNAi of a targeted isozyme gene, is often accompanied by abundance changes of a different subclass (or other sphingolipids of the same class). This implicates a regulatory mechanism that can sense and respond to sphingolipid changes.

## Materials and Methods

### *C. elegans* strains and maintenance

Worms were fed with *E. coli* OP50 on nematode growth medium plates and cultured at 20°C unless otherwise stated (42). The wild-type Bristol (N2) strain and mutant strain VC747 *lagr-1(gk327) I,* VC693 *cgt-1(ok1045) V,* RB1919 *sms-5(ok2498) II* were obtained from the the Caenorhabditis Genome Center. The double mutant *cgt-1(tm1027) V; cgt-2(tm1192) X* was kindly provided by Huanhu Zhu’s lab.

### Sample collection

To obtain synchronized animals, gravid adults were washed off plates with M9 buffer, then bleached with 30% sodium hypochlorite containing 0.75 M KOH to obtain the eggs. After hatching, the synchronized L1 worms were seeded onto fresh RNAi plates until harvest at young adult stage. Worms were washed three times with M9 buffer and pellet on ice; a total volume of 100 μl worm pellet was collected for each sample. For each RNAi conditions, three biological replicates were collected unless otherwise indicated.

### RNA interference

RNAi assays were performed at 20°C using the feeding method as previously described (43). All RNAi experiments are initiated from the L1 stage and ended at the young adult stage. The *Escherichia coli* strain HT115 transfected with L4440 (empty vector) was used as control. We employed available feeding RNAi library, the Ahringer library, and the Vidal library. All RNAi clones used in this study were verified by sequencing before use. The source of each RNAi clone is listed in Table 1.

**Table 1:** RNAi clones in this study

Target	RNAi source
*sptl-1*	Ahringer library
*elo-5*	Ahringer library
*hyl-1*	RCE library
*hyl-2*	Ahringer library
*lagr-1*	RCE library
*cgt-1*	Ahringer library
*cgt-3*	RCE library
*sms-1*	Ahringer library
*sms-2*	RCE library
*sms-3*	Ahringer library

### RNA extraction and qRT-PCR

Total RNA was extracted using the TRIzol reagent Invitrogen, Grand Island, NY) and the complementary DNA was synthesized using a reverse transcription kit (TAKARA, Kusatsu, Shiga, Japan). Quantitative PCR was carried out on a BioRad CFX96 real-time PCR system using a TAKARA real-time PCR kit (SYBR Premix Ex TaqTM II). *cdc-42* was used as the internal control. The qPCR primers are listed in Table 2.

**Table 2:** qPCR primers

Target	Forward	Reverse	Efficiency	R2
*cdc-42*	TCCACAGACCGACGTGTTTC	AGGCACCCATTTTTCTCGGA	91.2%	0.991
*pmp-3*	GTTCCCGTGTTCATCACTCA	ACACCGTCGAGAAGCTGTAGA	95.7%	0.987
*iscu-1*	GTCGCTTCAAATCAGTTCAGC	GTTCTTGTCAAGTGATCCGACA	100.6%	0.992
*sptl-1*	AGAGGAGTCACCGAGCACTTCA	AGTTGGGCATCAGCGGCTTTC	99.4%	0.991
*elo-5*	CATTGTTCTTCGCAAGAGACCACTT	GTAGCAGACAAGAGCATAGTAACCG	97.3%	0.994
*hyl-1*	GCAGGCTAAGGACGTCAGAT	CGGTGCTCGGAGATACCTTAT	92.50%	0.994
*hyl-2*	TGACCAGCAACCTCAAGCC	AGTATCGTAGGCGATCTTGAACA	96.50%	0.999
*lagr-1*	CCAGTGGGAATGCCAACCTA	AATACCAGACCAGCGAGCAC	94.90%	0.999
*cgt-1*	GTTTGCTTCTCAAGCTACCACT	ACTATACAACGTGGCAGGCAT	90.00%	0.995
*cgt-3*	TGAACAGTATGCAGAATGGCAA	AAGACGAAAGGAGCGGTGAG	101.20%	0.993
*sms-1*	TGGATCTATTGCAGACGACAA	CCGTCAGTCTCTTCGTGGAC	96.60%	0.999
*sms-2*	ACAGATGTGCTCCAGTCACG	TGTCTTCGCAGGTGAACTCT	108.7%	0.992
*sms-3*	TCGAATTGGGCAGTTTTGGC	AGCGCTACACAAAAGTCTCCTAA	91.90%	0.998

### Lipid extraction from *C. elegans*

The methyl-tert-butyl ether (MTBE)/methanol method was used for lipid extraction (44). After adding a fixed amount (0.3 μmol for each) of the internal standards (SM d18:1/12:0, Cer d18:1.10:0, GlcCer d18:1/12:0), worms were cryo-milled (Retch MM400). Two hundred and twenty-five microliters of methanol and seven hundred and fifty microliters of MTBE were added to the sample and incubated at 4°C for 10 min. Two hundred microliters of water was added and the mixture was centrifuged at 4,000 g for 8 min. The upper MTBE layer were collected and dried in the speed vacuum concentration system (Speed Vac). The extract was stored at −80°C and resolved in MeOH before analysis.

### LC-MS/MS analysis

LC-MS/MS analysis of sphingolipids was performed on a Thermo Vanquish UHPLC coupled with a Thermo Q Exactive HF-X hybrid quadrupole-Orbitrap mass spectrometer in positive ESI mode. The LC method was adapted from a published assay (45). Separation was carried out by a Waters ACQUITY UPLC BEH C18 column (1.7 μm, 2.1 × 100 mm). Column temperature was 60°C. Mobile phases consisted of water (A) and ACN: IPA 4:3 (B), both with 10 mM ammonium acetate and 0.1% formic acid. The following gradient was applied at a flow rate of 0.5 ml/min: 85% B (0–1 min), 85%–100% B (1–3 min), 100% B (3–8 min), 100%-85% (8–12 min), 85% B (12–15 min). Samples were resuspended with 200 μl methanol and 2 μl was injected.

Full-scan mass spectra were acquired in the range of *m/z* 100 to 1,200 with the following ESI source settings: spray voltage: 3.5 kV, auxiliary gas heater temperature: 380°C, capillary temperature: 320°C, sheath gas flow rate: 30 units, auxiliary gas flow: 10 units. MS1 scan parameters included resolution 60,000, AGC target 3e6, and maximum injection time 200 ms. MS2 scan parameters included resolution 30,000, AGC target 2e5, and maximum injection time 100 ms. Normalized collision energy was 30.

### Data processing and statistical analysis

Data preprocessing, including peak picking and alignment, was performed using MS-DIAL (46) software (v 4.16). An in-silico MS/MS library was developed for the annotation of ceramide (Cer), SM (SM) and GlcCer. First, theoretical *m/z* of sphingolipids with d17:1 sphingoid base and fatty acyls from C14 to C36 were calculated. Hydroxylated or unsaturated species were also included. After that, the fragmentation patterns of Cer, SM, and GlcCer were investigated from the experimental MS/MS spectra of reference standard compounds, for example, Cer d18:1/18:0, GlcCer d18:1/24:1, and SM d18:1/18:0. Here, the d18:1 species were used instead of d17:1 ones as the latter were not commercially available. Characteristic product ions and their relative abundances were used to predict the MS/MS spectra of d17:1 sphingolipids. For example, fragmentation of Cer d18:1/18:0 [M+H-H_2_O]^+^ yielded product ions at *m/z* 282.279 (C_18_H_36_NO^+^, 10% intensity of the base peak), 264.269 (C_18_H_34_N^+^, 100%), and 252.269 (C_17_H_34_N^+^, 10%), which originated from the C18 sphingosine (C_18_H_37_NO_2_). Therefore, replacing the C18 sphingosine with the C17 one gave the product ions of d17:1 Cer at *m/z* 268.264 (C_17_H_34_NO^+^, 10%), 250.253 (C_17_H_32_N^+^, 100%), and 238.253 (C_16_H_32_N^+^, 10%). Fragmentation pattern of GlcCer was basically the same as Cer. For SM, the only product ion was phosphorylcholine headgroup at *m/z* 184.073, regardless of their sphingosine and fatty acyl compositions. These fragment ions were combined with the calculated precursor *m/z* to generate the in silico library in MSP format using an in-house Java program. The MSP file was then converted to NIST lib format and searched against the experimental data with NIST MSPepSearch software. Hits with dot product score >600 were kept.

For quantitation, peak heights of the annotated sphingolipids were normalized with their corresponding internal standards. To compare the differences in each sphingolipid species between the RNAi group and the control group, Student's *t* test was performed. ∗*P* < 0.05; ∗∗*P* < 0.01; ∗∗∗*P* < 0.001; ns, *P* ≥ 0.5.

## Results

### A simple but effective LCMS-based workflow for the analysis of *C. elegans* sphingolipids

We started out by optimizing the method for analyzing *C. elegans* sphingolipids. As shown in Fig. 1B, for lipid extraction from cryo-milled *C. elegans* samples, we chose MTBE (44) method for ease of handing. Alkali depletion of glycerolipids (24, 47) is omitted because in our hands, it introduced greater variations without improving sensitivity. For chromatographic separation of sphingolipids, we tried hydrophilic interaction liquid chromatography (HILIC), which separates lipids by their polar headgroups, and reversed-phase liquid chromatography (RPLC), which separates lipids by fatty acyl chains. We opted for RPLC for two reasons: first, the performance of RPLC is more consistent than HILIC; second, ceramides tend to have poorly shaped chromatographic peaks when separated on a HILIC column and are thus difficult to quantify. After optimization of the LC gradient, we finally arrived at a 15-min C18 RPLC run that enables the separation of *C. elegans* sphingolipids (Materials and Methods and Supplemental Fig. S1).

From the high resolution, high mass accuracy MS/MS data, we only focused on sphingolipids built from the predominant id17:1 sphingoid base. For N-acyl chains, we considered fatty acids of 14–36 carbons with or without a hydroxyl group and with or without a double bond after an initial consideration of up to four double bonds. Cyclopropane fatty acids, which the worms acquire from their bacterial food mainly in the form of C19Δ and C17Δ (23), were in fact also considered because they are of the same mass as C19:1 or C17:1. However, we did not find C19Δ, C17Δ, or any unsaturated N-acyls in *C. elegans* sphingolipids.

From WT *C. elegans* staged to adult day 1, we identified a total of 54 sphingolipid species including 22 Cers, 10 GlcCers, and 22 SMs (Supplemental Fig. S1 and Supplemental Table S2), and 47 of them were quantifiable (Fig. 1C). As shown, *C. elegans* sphingolipids all have saturated N-acyl chains of 16–27 carbon atoms. The signal intensities of sphingolipids with hydroxylated N-acyl chains are much higher than those with non-hydroxylated N-acyl chains. In fact, all the GlcCers detected are hydroxylated. Our method cannot localize the hydroxyl group of the N-acyl, but based on the cumulative evidence provided by previous studies (19, 48, 49), it should be on C2 (Fig. 1A, inset, C22(OH) as an example). Among the ceramides, the highest MS signal intensity belonged to the C24(OH) species, followed by that of C22(OH). For GlcCers and SMs, the highest MS signal intensity species was found to be C22(OH) for both, followed by C24(OH) for GlcCers and C22 for SMs (Fig. 1C). The results above show that this simple, 15-min reversed-phase liquid chromatography-tandem mass spectrometry method enables effective profiling of *C. elegans* sphingolipids.

### Validation of the one-shot *C. elegans* sphingolipid analysis method

**Fig. 2:**
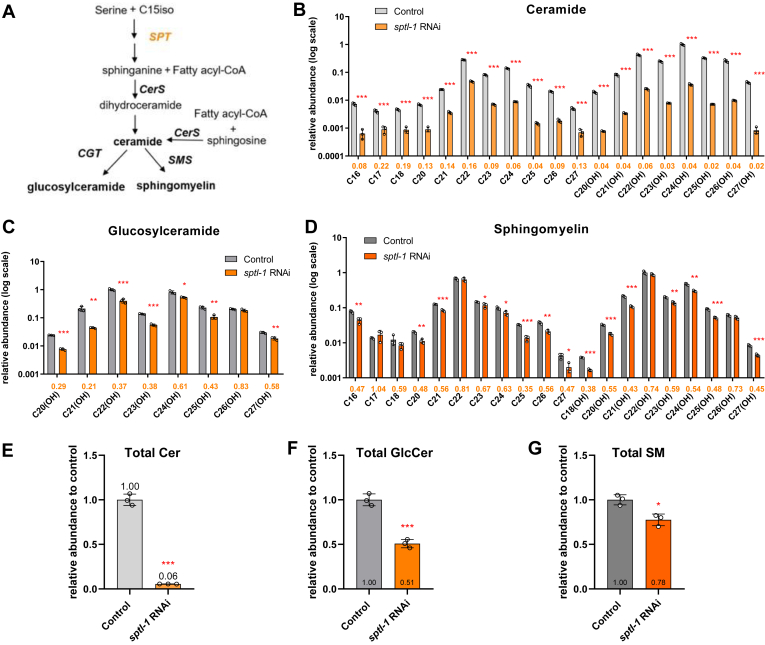
RNAi targeting the first key enzyme (SPT) in the de novo sphingolipid biosynthesis pathway caused a decrease of nearly all sphingolipids in *Caenorhabditis elegans*. A: A simplified diagram of de novo sphingolipid biosynthesis in *C. elegans*. *sptl-1* encodes the catalytic subunit of SPT in *C. elegans*. B–D: Relative abundance changes of individual Cer (B), GlcCer (C), and SM (D) species in *sptl-1* RNAi worms (log scale). n = 3 biological replicates, data are shown as mean ± standard deviation (∗*P* < 0.05; ∗∗*P* < 0.01; ∗∗∗*P* < 0.001). The colored values below each bar graph indicate the fold change of abundance of the RNAi-treated group versus the control group. E–G: Total Cer (E), GlcCer (F), and SM (G) level changes in *sptl-1* RNAi worms relative to the control RNAi worms. RNAi, RNA interference.

We validated our method on young adult *C. elegans* in which either the *elo-5* gene or the *sptl-1* gene was knocked down from the L1 stage by RNAi. RNAi reduced the mRNA level of *sptl-1* or *elo-5* by 60% or 80%, respectively (Supplemental Fig. S2). SPTL-1 is the *C. elegans* ortholog of the catalytic subunit of human serine palmitoyltransferase, which is located on the ER (26, 32). ELO-5 is a fatty acid elongase responsible for the biosynthesis of C15ISO (29, 50), which is a substrate required for the first step of a chain of reactions that lead to the formation of the id17:1 sphingoid base (Figs. 1A and 2A, and Supplemental Fig. S2A). RNAi of either *sptl-1* or *elo-5* is expected to reduce the sphingolipid levels in *C. elegans*, and this is indeed the case (Fig. 2 and Supplemental Fig. S2). In *sptl-1* RNAi worms, the total amount of Cer, GlcCer, and SM decreased by 94%, 49%, and 23%, respectively (Fig. 2E–G). With respect to individual sphingolipid species, *sptl-1* RNAi resulted in a significant decrease for nearly all of them except for GlcCer C26(OH), SM C22, and SM C26(OH) (Fig. 2B, D). RNAi of *elo-5* markedly lowered the levels of all ceramide species and most of the SM species (Supplemental Fig. S2B, D). Intriguingly, GlcCers, which activate mTOR in *C. elegans* and are required for larval development (50, 51), were least affected; only GlcCer C22(OH), the most abundant species in wild-type worms, had a significant, 66% decrease in *elo-5* RNAi worms (Supplemental Fig. S2C). This is in keeping with a recent study, which analyzed Cer and GlcCer but not SM, and showed that *elo-5* RNAi decreased GlcCer C22(OH) but increased GlcCer C21(OH) and C23(OH) (52). This result invites a notion that under sphingoid base deficiency, *C. elegans* might channel what is available to synthesize GlcCer as much as possible to best relieve a life-threatening condition. Taken together, the *sptl-1* RNAi and the *elo-5* RNAi results validated this simple but effective sphingolipid profiling method.

### Distinct sphingolipid profiles of RNAi mutants lacking one of the three ceramide synthase isozymes

Ceramides are synthesized on the ER by ceramide synthases (CerS) out of a sphingoid base and fatty acyl CoA. (Figs. 1A and 2A) (11). *C. elegans* has three CerS isozymes, HYL-1, HYL-2, and LAGR-1 (53, 54, 55). Previous studies have found that loss-of-function*(lf)* mutants of the CerS genes exhibited different phenotypes. For example, *hyl-1(lf)* worms are resistant whereas *hyl-2(lf)* worms are sensitive to anoxia stress (33); *hyl-1(lf)* and *lagr-1(lf)* suppressed radiation-induced germ cell apoptosis whereas *hyl-2(lf)* did not (34); L1 larvae of *hyl-1(lf)* or *hyl-2(lf)*, but not of *lagr-1(lf)*, are more susceptible to death under starvation (56).

**Fig. 3:**
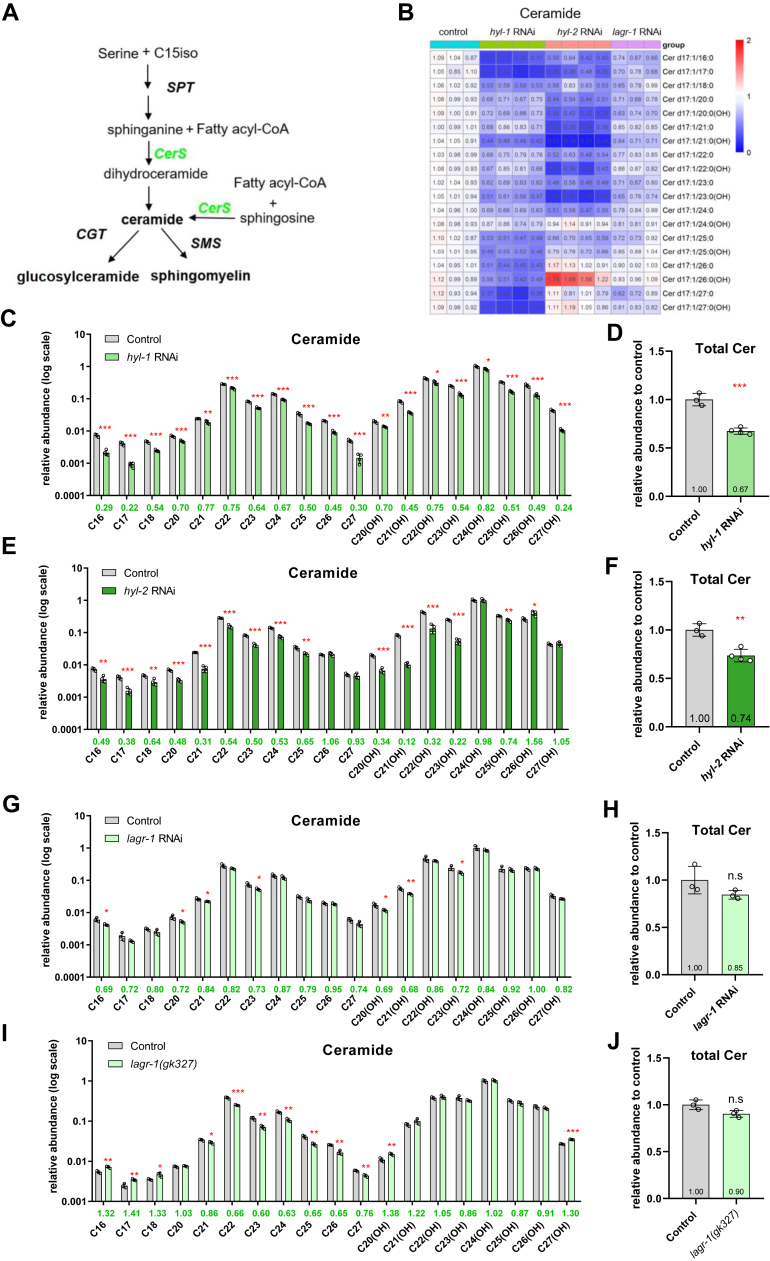
RNAi of each of the three ceramide synthase genes in *Caenorhabditis elegans* generated distinct ceramide profiles. A: In the *de novo* sphingolipid biosynthesis pathway in *C. elegans*, CerS catalyzes the formation of ceramides. B: A heatmap showing abundance changes of individual ceramide species in *hyl-1, hyl-2, lagr-1* RNAi worms relative to the control RNAi group. The average value of the three control RNAi samples, against which others were normalized, is set to 1. Abundance increase (fold change > 1) and decrease (fold change < 1) are indicated, respectively, by red and blue hues of varying saturation. C and D: Relative abundance changes of individual ceramide species (log scale) (C) and the total Cer level (relative to control) (D) in *hyl-1* RNAi worms. n = 3 biological replicates, data are shown as mean ± SD (∗*P* < 0.05; ∗∗*P* < 0.01; ∗∗∗*P* < 0.001). E and F: Relative abundance changes of individual ceramide species (log scale) (E) and the total Cer level (relative to control) (F) in *hyl-2* RNAi worms. G and H: Relative abundance changes of individual ceramide species (log scale) (G) and the total Cer level (relative to control) (H) in *lagr-1* RNAi worms. I and J: Relative abundance changes of individual ceramide species (log scale) (I) and the total Cer level (relative to control) (J) in *lagr-1(gk327)* worms. RNAi, RNA interference.

In this study, we found that *hyl-1* RNAi lowered the abundance of all ceramide species, and this was most pronounced for those containing hydroxylated or non-hydroxylated C25∼C27 N-acyl chains (decreased by 49–76%, Fig. 3B–D). Ceramides of C16∼C18 N-acyl chains also decreased to a similar extent upon *hyl-1* RNAi, but these species are of the lowest abundance in wild-type worms (Fig. 1C), so their decrease in absolute quantity is much less compared to the C25∼C27 species of high abundance.

In contrast to *hyl-1* RNAi, *hyl-2* RNAi decreased most noticeably the amount of ceramide species containing hydroxylated C20∼C23 N-acyls by 46–88%. Ceramide species containing longer (C24∼27), hydroxylated N-acyl chains were not or only marginally decreased by *hyl-2* RNAi, among which Cer C26(OH) actually increased significantly by 56% (Fig. 3B, E, F). These results are in agreement with the findings of an earlier study (33).

The sphingolipid profiles of the *lagr-1(lf)* mutant have not been analyzed before. Here, we found that *lagr-1* RNAi had only marginal effect on ceramide levels (Fig. 3B, G, H). Compared with *hyl-1* or *hyl-2* RNAi, *lagr-1* RNAi and *lagr-1(lf)* did not decrease the total amount of ceramide, and they affected only marginally a small subset of Cer species (Fig. 3G–J). These results suggest that HYL-1 and HYL-2 are the major ceramide synthases in *C. elegans* with differential preference toward N-acyl chains.

In addition to ceramides, sphingolipid profiling allowed us to examine the abundance of GlcCer and SM (Supplemental Fig. S3). Aside from decreasing the levels of ceramide species of VLCFAs (VLCFA, >24C), *hyl-1* RNAi also decreased the levels of GlcCer C27(OH) and five out of six VLCFA SM species, especially SM C27 and C27(OH) (Supplemental Fig. S3A, B, E, F).

In keeping with its effect on ceramides of hydroxylated or non-hydroxylated C20∼C23 N-acyls, *hyl-2* RNAi decreased the concentrations of four GlcCer species whose N-acyl chains varied from C20(OH) to C23(OH) (Supplemental Fig. S3A, C). Six out of eight SM species with hydroxylated or non-hydroxylated C20∼C23 N-acyl chains also decreased significantly in *hyl-2* RNAi worms (Supplemental Fig. S3E, G). Interestingly, *hyl-2* RNAi caused an increase of certain GlcCer and SM species, particularly the C24(OH) and C26(OH) varieties (Supplemental Fig. S3A, C, E, G).

Considering the modest effect of *lagr-1* RNAi on ceramides, with only 7 out of 19 Cer species displaying a weak but statistically significant decrease (by 16–32%), the effect of *lagr-1* RNAi on GlcCer and SM is disproportionally larger as it lowered the levels of 3 out of 8 GlcCer species (by 29–36%) and 13 out of 20 SM species (by 14–37%) (Fig. 3 and Supplemental Fig. S3). The total amount of SM was lower in *lagr-1* RNAi worms than the WT (Supplemental Fig. S3I) and possibly that of GlcCer, too, although in the latter case, the *P*-value (0.058) fell short of the 0.05 cutoff (Supplemental Fig. S3D, I).

### CGT-3 plays a major role in producing GlcCers compared to CGT-1/2

**Fig. 4:**
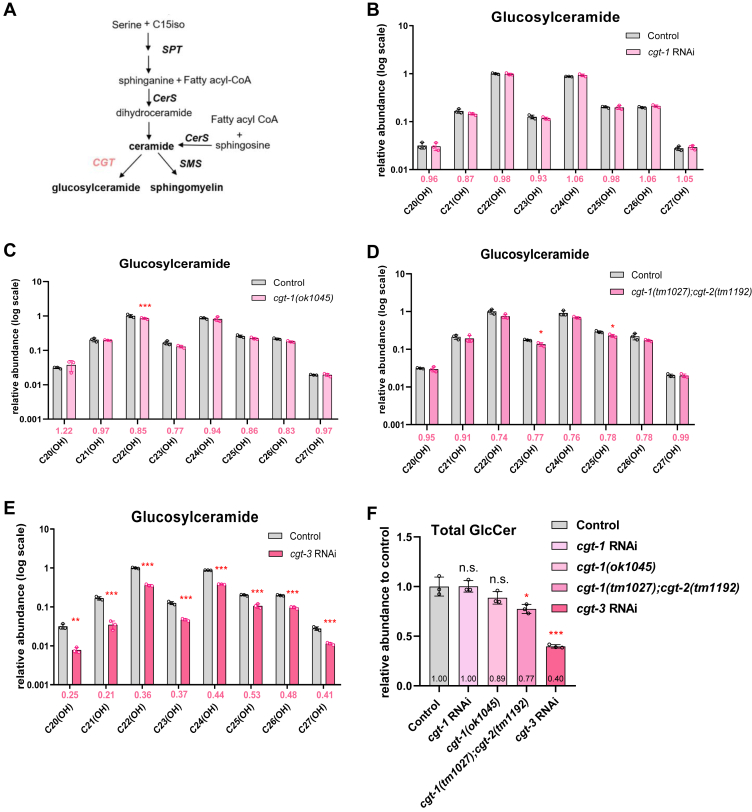
Deficiency of *cgt-3* but not *cgt-1* or *cgt-2* led to a large decrease of GlcCer levels. A: In the *C. elegans* sphingolipid biosynthesis pathway, CGT catalyzes the formation of glucosylceramides. B–E: Relative abundance changes of individual GlcCer species in *cgt-1* RNAi (B), *cgt-1(lf)* (C), *cgt-1;cgt-2(lf)* (D), *and cgt-3* (E) RNAi worms. n = 3 biological replicates, data are shown as mean ± SD (∗*P* < 0.05; ∗∗*P* < 0.01; ∗∗∗*P* < 0.001). The colored values below each bar graph indicate the fold change of abundance of the RNAi-treated group versus the control group. F: Total GlcCer levels in *cgt* RNAi or mutant worms relative to that in control worms. RNAi, RNA interference.

*C. elegans* GlcCers all have hydroxylated N-acyls. Synthesis of GlcCers is catalyzed by UDP-glucose:ceramide glucosyltransferase, for which *C. elegans* has three isozymes, CGT-1, -2, and -3. Mutations or RNAi treatments that compromise any single one of them did not show obvious L1 arrest phenotype (27, 57). However, *cgt-3;cgt-1* double mutant worms arrested and died at the L1 stage, *cgt-3;cgt-2* or *cgt-1;cgt-2* double mutants did not, and *cgt-2(lf)* did not further exacerbate the growth arrest phenotype of *cgt-3;cgt-1* worms (27, 57). Although there is consensus on the growth arrest phenotype of *cgt* mutants, there is none when it comes to GlcCer. *Nomura et al.* reported a marked decrease of total GlcCer in the *cgt-1* or *cgt-3* single mutant (27), but *Marza et al.* found no such effect (57). Both studies used TLC to quantify the total GlcCer level. Here, we analyzed individual GlcCer species by high-resolution mass spectrometry to dissect the functions of three CGTs. (Fig. 4 and Supplemental Fig. S4).

We found that *cgt-1* RNAi and a loss-of-function mutant *cgt-1(ok1045)* had little effect on GlcCer levels (Fig. 4B, C, F). In the *cgt-1; cgt-2* double mutant, only a slight decrease was detected for two GlcCer species (Fig. 4D). In contrast, *cgt-3* RNAi reduced the total GlcCer level by 60% and every single GlcCer species quantified by 47–79% (Fig. 4E, F). These results demonstrate that CGT-3 is the major enzyme for GlcCer production among three CGTs.

Additionally, *cgt-3* RNAi caused a decrease of all the ceramide species with hydroxylated N-acyl chains by 37–57% (Supplemental Fig. S4C, D). In other words, when the activity of CGT-3 is compromised, both the substrate (Cer with 2-OH N-acyl chain) and the product (GlcCer with 2-OH N-acyl chain) are reduced, instead of a product decrease leading to a substrate buildup. This could suggest a feedback mechanism that keeps the substrate and the product levels in balance. SMs were not affected by *cgt-3* RNAi in a marked way (Supplemental Fig. S4G, H).

### Overlapping sphingolipid profiles of RNAi mutants lacking a SM synthase SMS -1, −2, or −3

*C. elegans* SM synthases SMS-1, -2, and -3 are homologs of human SMS2, which is located at the PM as well as the Golgi. Human SMS2 is a membrane protein and its catalytic site faces the extracellular space or the Golgi lumen (40, 58). SMs are synthesized de novo in the Golgi, and those in the outer leaflet of the PM could be remodeled through the actions of acid SMases and SM synthases. Much is unknown about the functions of *C. elegans* SMSs other than that *sms-1(lf)* suppressed clozapine-induced developmental delay or lethality, whereas *sms-2(lf)* or *sms-3(lf)* did not (59).

**Fig. 5:**
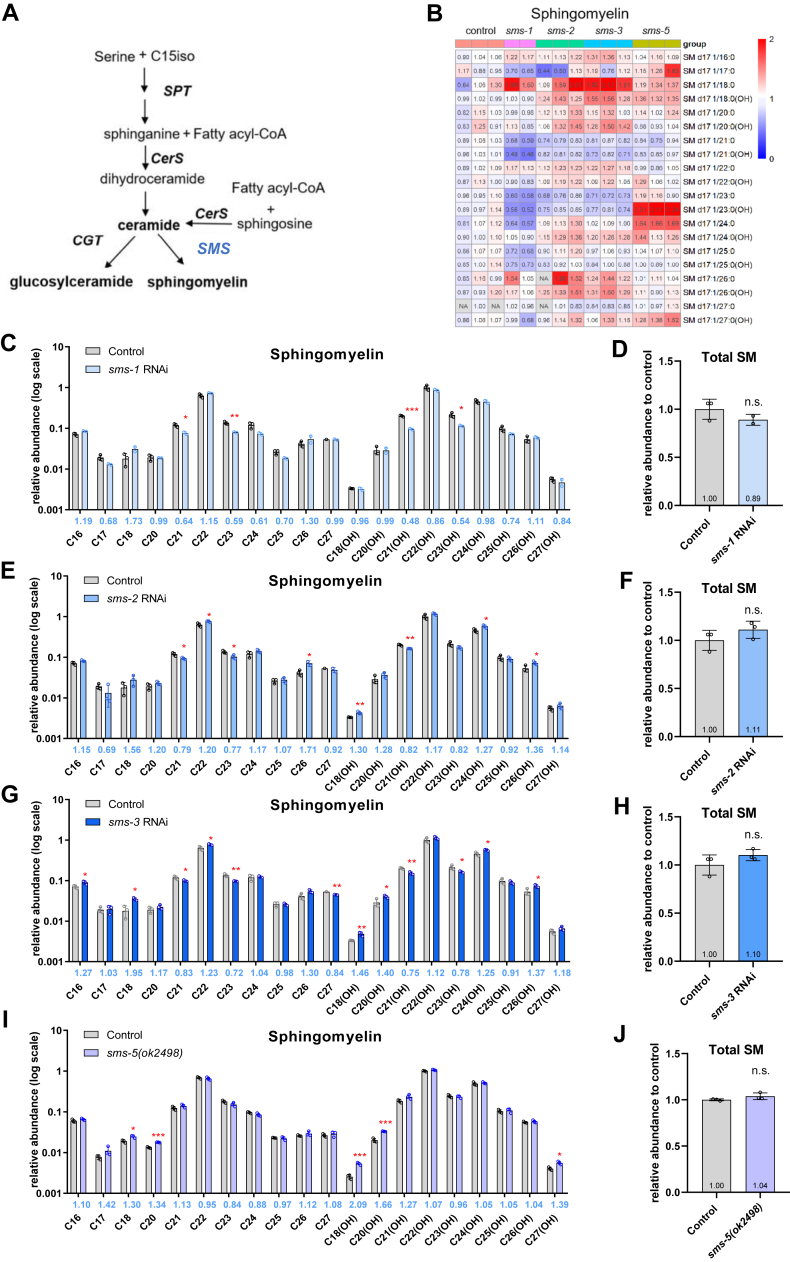
RNAi of *sms-2* or *sms-3* generated similar SM profiles, which differed from that by RNAi of *sms-1* or deletion of *sms-5*. A: In the *C. elegans* sphingolipid biosynthesis pathway, SMS catalyzes the formation of sphingomyelins. B: A heatmap showing abundance changes of individual sphingomyelin species in *sms-1, sms-2, sms-3* RNAi worms and *sms-5(ok2498)* relative to the control. The average value of three control samples, against which others were normalized, is set to 1. Abundance increase (fold change > 1) and decrease (fold change < 1) are indicated, respectively, by red and blue hues of varying saturation. C and D: Relative abundance changes of individual SM species (log scale) (C) and the total SM level (relative to control) (D) in *sms-1* RNAi worms. n = 3 biological replicates, data are shown as mean ± SD (∗*P* < 0.05; ∗∗*P* < 0.01; ∗∗∗*P* < 0.001). E and F: Relative abundance changes of individual SM species (log scale) (E) and the total SM level (relative to control) (F) in *sms-2* RNAi worms. G and H: Relative abundance changes of individual SM species (log scale) (G) and the total SM level (relative to control) (H) in *sms-3* RNAi worms. I and J: Relative abundance changes of individual SM species (log scale) (I) and the total SM level (relative to control) (J) in *sms-5(ok2498)* worms. RNAi, RNA interference.

In this study, we found that knockdown of either *sms-1, -2,* or *-3* resulted in a slight decrease of several SM species, especially those with odd-chain fatty-acyl groups but none affected the total SM level (Fig. 5). The *sms-1* RNAi worms had a SM profile that is different from that of *sms-2* or *sms-3* RNAi worms (Fig. 5). As shown, *sms-1* RNAi caused a 30–52% decrease of SM species whose N-acyl groups, hydroxylated or not, contain an odd number of carbon atoms such as C21, C23, or C25 (Fig. 5B, C), which are presumably mmBCFAs. One even-numbered N-acyl SM species (SM C24) decreased by 39% in *sms-1* RNAi worms but it was not statistically significant (Fig. 5B, C). For *sms-2* RNAi and *sms-3* RNAi, although they lowered the quantities of four SM species containing an N-acyl group of odd numbered carbons (C21, C23, and their hydroxylated forms)—somewhat similar to *sms-1* RNAi—they elevated the levels of several SM species with even-numbered N-acyl chains such as C22, C24(OH), and C26(OH), which was not evident under *sms-1* RNAi (Fig. 5B, E, G). In brief, our data suggest that *sms-1, -2,* and *-3* all contribute to the production of SM species containing a C21, C23, C21(OH), or C23(OH) N-acyl group (Fig. 5), which are of medium intensity (Fig. 1C). Further studies are needed to find out whether SMS-1, -2, and -3 are redundant for producing high-abundance SMs with even-numbered N-acyls.

In contrast, *sms-5(ok2498)*, a putative null allele of the *C. elegans* homolog of human SMS1, did not decrease the level of any SM species (Fig. 5B, I), possibly because the expression level of *sms-5* is the lowest among these SMSs (Supplemental Fig. S6A). On the contrary, for five minor SM species, deletion of *sms-5* increased their levels. Displayed in Supplemental Figs. S5 and S6 are additional findings from the sphingolipid profiling experiments. *1) sms-2* and *sms-3* RNAi lowered the levels of nearly all ceramide species containing an odd-chain N-acyl group to a larger extent than *sms-1* RNAi (Fig. 5A and Supplemental Fig. S5A–D). *2)* Similar trend was observed when it comes to the abundance of GlcCer C21(OH) (Supplemental Fig. S5F–I). *3) sms-1* RNAi increased the levels of three GlcCer species C24(OH), C26(OH), and C27(OH) to a larger extent than *sms-2* or *sms-3* RNAi (Supplemental Fig. S5F–I). *4) sms-5(ok2498)* caused a slight decrease of eight non-hydroxylated Cer species (Supplemental Fig. S6B).

## Discussion

For a biosynthetic pathway of the simplest type, one would expect that following the inactivation of an enzyme, the product of the reaction catalyzed by that enzyme as well as other metabolic products further downstream will deplete, while the substrate and other precursor metabolites further upstream will accumulate. This is not the case for sphingolipid biosynthesis as can be seen from the sphingolipid profiles of *cgt-3* RNAi worms and *sms-2* or *sms-3* RNAi worms. The decrease of GlcCer as a direct consequence of *cgt-3* RNAi was accompanied not by an increase but a decrease of the ceramide substrate (Fig. 4 and Supplemental Fig. S4). The lack of an increase could not be explained by surplus ceramides being used to make more SMs, as the total SM level hardly changed despite small increase or decrease of certain species (Supplemental Fig. S4). Likewise, under *sms-2* or *sms-3* RNAi, the decrease of SM C21/23 and SM C21/23(OH) was accompanied not by an increase but a decrease of the ceramide substrate (Fig. 5 and Supplemental Fig. S5). Again, the lack of an increase was not because surplus ceramides were used to make more GlcCer (Supplemental Fig. S5). Hence, the sphingolipid biosynthesis pathway is not a simple one; it appears to be under strict regulation to prevent an overabundance of ceramides when a downstream biosynthesis step is obstructed. In other words, the ceramide levels need to be kept in balance with those of GlcCer and SMs.

The sphingolipid profiles of the *sms-2* and *sms-3* RNAi worms revealed an interesting phenomenon (Fig. 5). The decrease of SMs C21, C21(OH), C23, and C23(OH) was accompanied by an increase of SMs C22, C24(OH), and C26(OH). Similarly, in *elo-5* RNAi worms, the decrease of GlcCer C22(OH) was accompanied by an increase of GlcCer C21(OH) and GlcCer C23(OH) (Supplemental Fig. S2). This suggests that there may be a mechanism that could upregulate certain sphingolipids of even-numbered N-acyl chains to compensate a decrease of sphingolipids of odd-numbered N-acyls and vice versa. We have noticed that across the RNAi samples examined in this study, the abundance changes of GlcCer C23(OH), SM C21, and SM C21(OH) anti-correlated with most other sphingolipids including 16 out 21 species with even-numbered N-acyls (Supplemental Fig. S7). In keeping with this, the abundance changes of GlcCer C26(OH) and SM C26(OH) anti-correlated with Cer C21/C21(OH)/C23(OH), GlcCer C23(OH), and SM C21/C23/C21(OH), all with odd-numbered N-acyls (Supplemental Fig. S7). We thus propose that sphingolipids of even-numbered N-acyls may be regulated separately—at times oppositely—from those of odd-numbered, presumably mmBCFA N-acyls.

In this study, we identified from young adult *C. elegans*, a total of 54 species out of three classes of sphingolipids Cer, GlcCer, and SM. In Supplemental Table S1, the sphingolipid identification results from this study are compared with those from previous ones. It is evident that the high-abundance Cer, GlcCer, and SM species were identified consistently across studies. The absence of GlcCer species with non-hydroxylated N-acyl chains, first reported in 1995 (19), was later verified by four studies including this one (24, 39, 47) and countered by one study (25). This comparison shows that the sphingolipid profiling method developed here is sensitive and accurate, and it enables comprehensive analysis of *C. elegans* Cer, GlcCer, and SM.

## Data Availability

The raw lipidomics data from HPLC/MS-MS measurements are available upon request.

## Supplemental data

This article contains supplemental data (19, 24, 25, 39, 47).

## Conflict of interest

The authors declare that they have no conflicts of interest with the contents of this article.
